# Prospective cohort study to assess rates of contagious disease in pre-weaned UK dairy heifers: management practices, passive transfer of immunity and associated calf health

**DOI:** 10.1136/vetreco-2017-000226

**Published:** 2017-11-28

**Authors:** Kate F Johnson, Natalie Chancellor, Charlotte C Burn, D Claire Wathes

**Affiliations:** Department of Pathobiology and Population Sciences, Royal Veterinary College, Hatfield, UK

**Keywords:** calves, dairy cattle, diarrhoea, health, respiratory disease, passive immunity

## Abstract

Dairy calves are vulnerable to infectious diseases, particularly diarrhoea and bovine respiratory disease (BRD), causing mortality and reducing welfare and growth. A prospective cohort study was performed on 11 UK dairy farms to determine the underlying causes for calf disease. This first paper describes the incidence, timing and duration of infectious disease, mortality rates, passive transfer of immunity and key management practices that may contribute to disease incidence. Heifer calves were recruited in the first week of life (n=492) and a blood sample taken to measure IgG and total protein (TP). Each animal was examined weekly for nine weeks using a standardised health scoring system. Recruitment of calves occurred between August and February. Four farms provided supplementary colostrum to more than 75 per cent of calves born, whereas on the remainder only 0 to 19 per cent were supplemented. Mean serum IgG and TP were 19.0±10 and 56.7±10.3 mg/ml respectively, with 20.7 per cent (95CI: 17.2 to 24.7 per cent) of all calves classified as having failure of passive transfer (IgG <10 mg/ml). The overall preweaning mortality rate was 4.5 per cent. (95 per cent CI: 2.9 to 6.8 per cent). During this period,48.2 per cent of all calves (range 24.1 to 74.4 per cent between farms) were diagnosed with diarrhoea and 45.9 per cent (range 20.4 to 77.8 per cent) with BRD. The incidence rates were 7.8 cases of diarrhoea and 10.1 cases of BRD per 100 calf weeks at risk, respectively. Rates of infectious disease were therefore high despite relatively good passive transfer.

## Introduction

Most mortality and contagious disease in dairy heifers occurs during the milk feeding period.^1–3^ Reported mortality rates in young calves (up to age two–six months depending on the study and excluding perinatal losses) were between 2.4 per cent and 11.7 per cent, with diarrhoea and bovine respiratory disease (BRD) listed as the major causes in this age group (reviewed by Johnson and others).^4^ Death rates of calves on individual farms within studies varied widely from 0 to 30 per cent. As some farms were able to achieve less than one per cent overall mortality, this suggested that deaths on other farms may be preventable with improved husbandry using existing strategies.^5 6^

Calf mortality is also indicative of underlying problems. Sick animals have poor welfare and are unable to express normal behaviours.^7^ There are associated veterinary expenses and an economic cost of reduced growth rates.^8^ Juvenile disease may compromise development in surviving heifers and prevent them from reaching rearing targets.^9–11^ For example, first parity was delayed by a median of six months in heifers that had BRD in the first three months of life^12^ and calves that had four episodes of BRD before first calving had 1.87±0.14 greater odds of failing to complete their first lactation compared with healthy calves.^13^ Such animals are also unlikely to recoup their rearing costs.

BRD and diarrhoea can be diagnosed and recorded by several different systems, and several studies have shown that this can influence the disease rates reported. EU farmers must keep a medicines book, although this does not include rehydration treatments for diarrhoea. Comparisons between farmer diagnosis with weekly clinical examinations by veterinarians showed only moderate agreement, with Sivula and others^14^ reporting a kappa statistic of 0.47 with respect to BRD. This is a test of interobserver agreement, measured on a scale of 0, chance to 1, perfect agreement. Virtala and others^10^ found that pneumonia diagnosed by a veterinarian was significantly associated with a reduced bodyweight gain in calves (P<0.01), whereas this relationship was not significant when farmer diagnosis was used instead (P=0.47). They attributed this difference to false-positive diagnoses by the farmer. On the other hand, only 47 per cent of cases of respiratory disease detected by farmers were treated with antibiotics, highlighting frequently missed treatment opportunities.^15^ These findings call into question the reliability of disease diagnostic information supplied by producers. The format in which data are reported can also vary. Cumulative incidence, defined as the total number of new cases during the study period, is widely used. This does not, however, account for duration of disease and repeated cases in an individual may be unrepresented.

A further challenge for effective treatment is identification of the causative agent. Calf diarrhoea and BRD are not caused by single pathogens but are generally composite syndromes where clinical signs depend on pathogens, host and environment and their interactions.^16^ Consequently, focusing on clinical state rather than the putative pathogen can be an effective strategy to identify disease problems in both clinical and research contexts.^1 14 17^ Many aspects of calf housing also impact on calf health. These include building design (ventilation, temperature, humidity, etc), cleanliness of the pens and feeders, pen size and proximity to other calves and older cows.^18 19^ Receiving an adequate supply of good quality colostrum within the first 12 hours after birth is considered essential for calf health by providing passive immunity.^20^ After ingestion, IgG is transferred to the calf’s circulation, whereas IgA acts locally within the intestinal mucosa.^21^ Calves receiving inadequate colostrum experience a failure of passive transfer (FPT), which is assessed by measuring circulating levels of IgG and/or total protein (TP). Values less than 10 mg/ml IgG or less than 50 mg/ml TP have generally been defined as the cut-off points for FPT, although others have used 12 and 55 or 57 mg/ml, respectively.^22–26^ Given that contagious diseases affecting calves have a multifactorial nature, they are influenced by a dynamic and varied group of factors. However, accurate health information about dairy heifers on the UK commercial farms is currently lacking, with recording of calf disease and mortality being much lower than in Scandinavian countries.^27^ Youngstock are rarely included in computer-based recording systems used for the adult herd and paper-based records are often incomplete or inaccurate. Only 39 per cent of Welsh farmers in a recent survey recorded calf disease, with veterinarians describing records as adequate on only 8 per cent of farms for diarrhoea and 16 per cent for respiratory disease.^28^ Farmers are consequently often unaware of reliable disease or death rates for their own calves. The UK is a significant dairy-producing country with around 1.9 million adult dairy cows^29^ and consequently this reflects a significant gap in existing knowledge.

The aim of this prospective cohort study was to monitor preweaned heifers on commercial farms with weekly health visits to assess their health and passive transfer status and to determine whether areas of the UK dairy heifer management require improvement. The evidence on farmer recording cited above suggests that trained technicians or veterinarians are required to ascertain disease rates reliably. This is the first in a series of four papers and provides a description of the cohort, key management practices, measures of passive transfer and disease incidence. Future papers will address the factors associated with disease risk in individual calves, calf growth rates and aspects of colostrum management.

## Materials and methods

### Cohort design and recruitment

A convenience sample of 11 dairy herds in S.E. England were recruited by approaching local veterinary surgeries to suggest farmers to participate. Farms were then visited to discuss the project and complete a consent form. For inclusion in the study, farms needed to expect 30–50 heifers to be born during a recruitment period of four months and ranged in size from 186 to 550 adult cows. There were two calf recruitment periods, either October 2011 to February 2012 (seven farms) or August to October 2012 (four farms). During these times, all births were recorded and all heifer calves alive after 24 hours were recruited at weekly visits. A literature review revealed that while little was known of calf disease rates in the UK, the incidences of diarrhoea and BRD were usually between 10–25 per cent and 7–29 per cent, respectively in other countries which have included the USA, Canada, Holland and Sweden.^4^ Assuming a rate for each of these diseases of around 20 per cent, a target cohort of 500 was considered adequate to allow for subsequent comparisons between sick and healthy animals.

### Herd profiles including management practices

Information on herd details and calf rearing practices for each farm were collected. This initially involved an interview with the farmer or herdsmen using a standardised data capture template as described by Brickell and others.^30^ These farm records were subsequently updated and additional information on the individual calves was obtained during the weekly data collection visits. Information on the farm included herd size (number of adult cows, number of heifers being reared), calving pattern, predominant breeds kept, ownership and staffing and average milk yield. Calving information included where the cows calved (group paddock, straw yard, individual pen) and age of separation of calf from dam (snatched, 12–24 hours, or left with dam for >24 hours). For each live heifer calf recruited onto the study, data were obtained on whether dystocia occurred on a 3-point scale (1, no assistance; 2, mild pull; 3, traction). Routines for calf housing on each farm and group sizes were recorded and it was noted if the calves were transferred to another pen before weaning. If so, the age at which this occurred and whether or not it involved mixing calves from different initial groups was recorded. Details of vaccination policy for preweaned calves were obtained. Water availability was noted at each visit, including issues with spilling and fouling that prevented adequate access to palatable water.

### Measurement of passive transfer

Farms recorded which calves received supplementary colostrum by bottle or tube. A jugular blood sample was taken into a plain Vacutainer (BD, Oxford, UK) at the first clinical examination, when calves were between one and eight days old. These samples were collected under the UK Animals (Scientific Procedures) Act 1986,^9^ under project licence PPL 70/7223, also approved by the RVC Ethical Review Process. After clotting, the sample was centrifuged and the serum stored at −18^o^ C for later analysis. A refractometer (RHC-200, Linx, China) was calibrated with distilled water then used to assess serum TP. IgG was measured using a radial immunodiffusion assay kit (VMRD, Pullman, Washington, USA) according to the manufacturer’s instructions. After 18–24 hours incubation at room temperature, each gel was scanned using a gel reader (G:Box, Syngene, Cambridge, UK) and the diameter of each precipitate ring was then measured using ImageJ (https://imagej.nih.gov/ij/).

### Collection of health data

Farmers were asked to record details of calving and calf disease on preprinted white boards and this information was checked at each weekly visit. Calving information was well recorded but disease information was not added consistently so could not be included in the analysis. Where farmers were already treating an animal, their presumed diagnosis was recorded and confirmed at the next clinical examination. The clinical scoring system for calf health was developed by the University of Wisconsin-Madison and used a systematic approach to assess faecal consistency and signs of BRD.^1^ Some minor modifications were made for use in the UK as summarised in Table 1. Several areas were adopted with no changes: ocular and nasal discharges and presence of induced or spontaneous cough were scored on a scale from 0 to 3. Head tilt, a sign typical of inner ear infection, was excluded because it was not seen in any calf in the study. Temperature scoring used cut -off values of 38.5^o^C, 39^o^C and 39.5^o^C (rather than 101^o^F, 102^o^F and 103^o^F). Animals with a respiratory score ≥5 were classified as having BRD. Faecal scoring was used as described in Table 1 and those calves with a faecal score greater than and equal to 2 were classified as having diarrhoea. Notes were also taken at each visit to record any other problems, most commonly umbilical infection. Clinical examinations were completed on every calf each week for nine consecutive weeks by the same research veterinarian.

**TABLE 1:** Health scoring system used in the weekly clinical examinations, adapted from McGuirk^1^ for use in the UK

	Calf health scoring criteria for respiratory disease			
	0	1	2	3
Rectal temperature	<38.5^o^C	38.5–38.9^o^C	39.0–39.5^o^C	>39.5^o^C
Cough	None	Induced single cough	Induced repeated coughs or occasional spontaneous cough	Repeated spontaneous coughs
Nasal discharge	Normal serous discharge	Small amount of unilateral cloudy discharge	Bilateral, cloudy or excessive mucus discharge	Copious bilateral mucopurulent discharge
Eye scores	Normal	Small amount of ocular discharge	Moderate amount of bilateral discharge	Heavy ocular discharge
Total respiratory score ≥5 classified as having bovine respiratory disease				
Faecal score	Normal	Semi-formed, pasty	Loose but stays on top of bedding	Watery, sifts through bedding
Faecal score ≥2 classified as having diarrhoea				

For ethical reasons, both the farmers and their local veterinary surgeon were informed after each visit of any animals showing clinical signs of disease. Each farm had agreed treatment protocols in place. For BRD, this always included a licensed antibiotic and a non-steroidal anti-inflammatory drug. In the UK, these must legally be recorded in a medicines book; these books were checked weekly to determine which calves had been treated for BRD. No farmers kept any calf disease records other than their medicines books. For diarrhoea, the treatment protocol used rehydration solutions which did not legally have to be recorded. In practice it was not, therefore, possible to obtain exact information about which calves were fed rehydration solution or how many times they were treated. Where farmers described having treated calves but there were no signs of any disease on clinical examination, the clinical signs recorded by the farmer were used. All calf deaths were recorded and the presumed cause was established by the local veterinarian by means of a gross pathology postmortem examination.

### Data analysis

Data were stored in Excel and arranged into a relational database using Access to allow for further analysis (Microsoft Office, Microsoft, Redmond, Washington, USA). Missing data were stored as an ‘NA’ for not available and no interpolation was used. All analysis was completed in R using the lattice package for graphics (http://www.r-project.org). The passive transfer measurements were normally distributed, so these results are presented as mean (±sd). Other data on mortality, disease and colostrum management were count data and results are presented as the count and the percentage. To calculate incidence rates for disease, the number of new cases was divided by calf weeks at risk. New cases were defined as a calf meeting the diagnostic criteria that had been scored as healthy for that condition in the previous week. For incidence rate calculations, this meant that repeated cases were included but continuing disease was not. The calculations on how many weeks a calf had a disease used the total number of positive clinical examinations and did not account for difference between long disease duration compared with repeated cases. CIs to compare proportions of animals affected between farms were calculated using the test of equal proportions (prop.test). Differences between farms were assessed by analysis of variance (ANOVA) with post hoc testing using the Tukey HSD method in the R package ‘agricolae’ to correct for multiple testing. A Student’s *t* test was used where there were only two groups for comparison. The level of significance was set at P<0.05.

## Results

### Herds and breeds

Three of the 11 farms (A, H, K, Table 2) had an autumn block calving pattern (September to November), with the remainder having all-year-round (AYR) calving. Five farms were pure Holstein, three were Holstein with some additional Viking Red crosses, and one farm had Holsteins alongside Ayrshires. Of two farms using more grass-based systems, one kept predominantly New Zealand Friesian type animals along with some Viking Red cross Friesian and the other had a large number of mixed breed cattle with many Jersey and Jersey crosses alongside Friesian cross heifers. From these farms, 492 dairy heifer calves were recruited, with a range of 26–56 calves per farm (Table 2). In total, 351/492 (71 per cent) of these calves were Holsteins with the next largest groups being black and white cross Viking Red cattle (43/492, 8.7 per cent) and Friesians (37/492, 7.5 per cent). Of the 470 calves that survived until the nine-week clinical examination, 403 had a complete data set (85.7 per cent), 45 had a single missing data point (9.8 per cent) and 22 calves (4.5 per cent) had more than one week of missing data.

**TABLE 2:** Summary of calf management practices for the 11 farms included in the study

Farm	A	B	C	D	E	F	G	H	I	J	K
Number of cows	247	300	280	550	320	450	190	350	550	186	240
Number of calves	54	36	48	51	48	39	35	54	56	26	45
Breed	Mixed*	H	H/VRX	F/VRX	H/VRX	H	H/A	H/VRX	H	H	H
Calving area	GP	SY	SY	SY	SY	IP†	IP	GP	SY	SY‡	GP
Group size (1)	10–20	1	3	1	3	5	1	10–20	5	1	5
Age at transfer	3–5 d	1–2 d	1 w	1 w	1 w	3 w	2–3 w	3–5 d	1 w	NA	1–2 w
Group size (2)	30	1	4–6	12	4–6	20	3	30	30–45	NA	15
Feeding method	Auto	Bucket	Drum	Bucket	Drum	Auto	Bucket	Auto	Auto	Bucket	TF
Feeds/d	NA	2	NA	2–1	NA	NA	2–1	NA	NA	2	2
Type of milk	MR	MR	Waste	MR	Waste	MR	MR	MR	MR	MR	MR
Forage feed§	Hay	Straw§	Silage	Straw	Silage	Straw	Straw	Hay	Straw	Straw§	Straw§
Weaning period	1 w	1 w	2 w	1 w	2 w	1 w	1 w	1 w	1 w	1 w	2 w
Weaning age	8–9 w	6–8 w	7–9 w	7 w	7–9 w	8–10 w	6–9 w	8–9 w	8–9 w	6–8 w	7–8 w
Vaccines	No	Yes¶	Yes¶	No	No	No	No	No	No	No	No

*Jersey, FriesianxJersey, Jerseyxother, Friesianxother.

†Calves snatched as soon as observed.

‡Age at separation from dam between 12 hours and several days.

§Straw only provided as bedding.

¶Intranasal Rispoval (Zoetis UK, Tadworth, UK) preweaning for respiratory syncytial virus and parainfluenza.

A, Ayrshire; d, day; F, Friesian; GP, group paddock; H, Holstein; IP, individual pen; MR, milk replacer; NA, not applicable; SY, straw yard; TF, teat feeder; VRX, Viking Red cross; w, week.

### Preweaning feeding and housing management

The management practices for calving and calf management are summarised in Table 2. The three autumn calving herds calved outside in group paddocks. Six herds calved in groups in straw yards. Two farms used individual calving pens, with one of those snatching calves soon after birth as a Johne’s disease control strategy. Most other farms left calves with their dams for an estimated 12–24 hours, although this was highly variable and some individual calves on one farm remained with their dams for up to 10 days.

The initial group size ranged from single pens up to groups of 20 calves. Four farms fed calves using automated feeders. Three of these calves were moved from the farm of origin to a nearby rearing unit once passports were issued (typically at one week old). The other kept calves at the farm of origin and bucket fed twice daily for approximately three weeks until a batch was moved to a rearing unit. Three farms fed milk twice daily, two using buckets and keeping calves in single pens and two using feeders with teats and keeping calves in groups of 12. A further two farms fed calves twice daily for approximately two weeks and then switched to once-daily milk feeding. This study was completed in 2012, before the clarification issued by the Department for Environment, Food and Rural Affairs (DEFRA) that once-daily feeding under 28 days of age was impermissible.^31^ Finally, two farms kept calves in small groups where there was a single teat per pen of four to six calves supplied by a mixed drum of acidified waste milk. All farms provided forage and commercial calf pellets fed ad libitum from the first week of life until after weaning. All farms bedded on deep straw: on three farms this was the only source of fibre, four farms fed additional chopped straw off the ground, two fed hay and two offered silage to calves. No farmers provided water in the pens used to house calves for the first few days. All farms then provided water, although there were regular issues with spilling and fouling that often prevented adequate access to palatable water. Farm F did not provide water premovement to their rearing unit, which occurred at an average age of 29 days.

**FIG 1:**
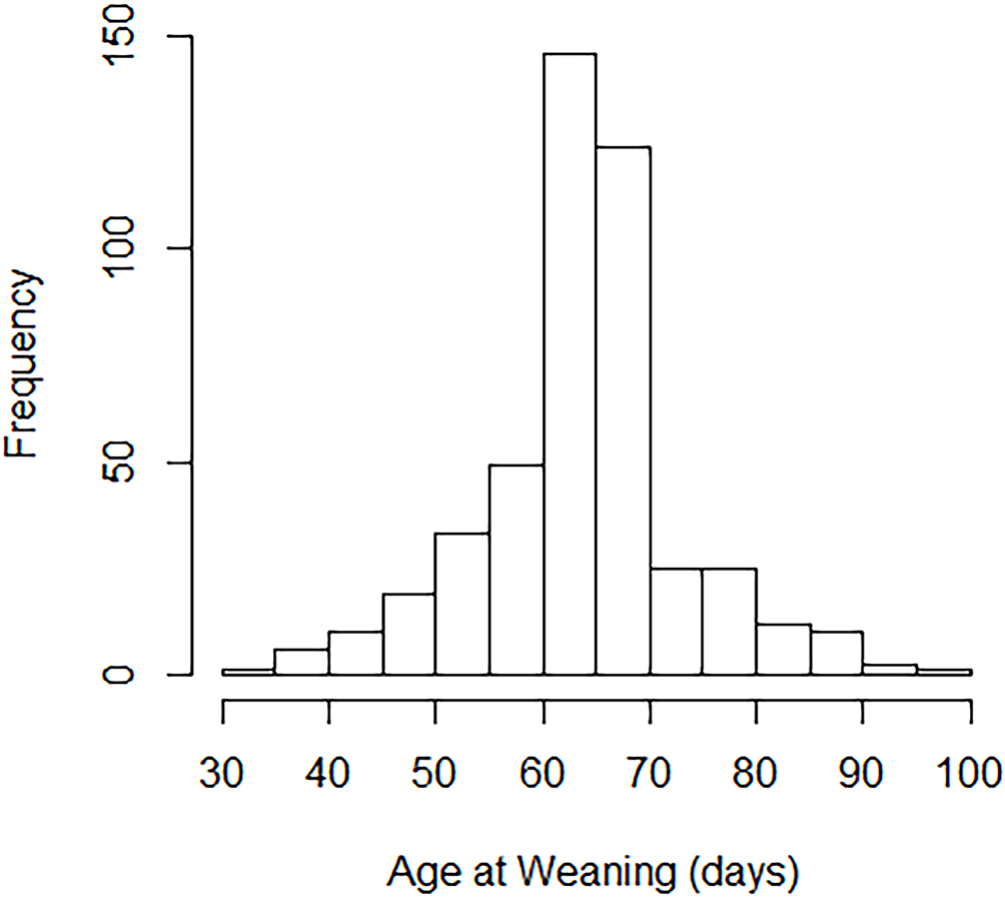
Histogram illustrating range of weaning ages for the 492 calves in the study.

All farms used gradual step-down weaning, typically over 1 week beginning at 6–10 weeks of age. Most farms based their timing decision on a combination of calf age, calf or group appearance and convenience. The interquartile range for weaning age was quite narrow, with half of all calves weaned between 60 and 69 days of age, but the overall range from 34 to 96 days was much wider (Fig 1).

### Colostrum feeding management and passive transfer

No farms monitored either colostrum quality or passive transfer outside the study. The supplemental colostrum used was previously frozen from the first milking and generally fed at 2×2 litres. No farms used artificial colostrum supplements. Farm F pasteurised colostrum as a Johne’s disease control measure. The two largest AYR calving farms routinely fed supplemental colostrum using either an oesophageal tube or bottle (Table 3). Two further farms gave supplementary colostrum to most calves (75 per cent and 84 per cent, respectively). The remaining seven farms supplemented when staff felt it was required, but on these farms only between 0 per cent and 19 per cent of calves received any additional colostrum during the study period. In total, 35.2 per cent (173/492) of calves received supplementary colostrum.

**TABLE 3:** Details of passive transfer by farm as measured in the first week of life*

Farm	n calves	% supplemented	IgG (mg/ml)	Total protein (mg/ml)	n (%) calves with failure of passive transfer	
					IgG <12 (mg/ml)	IgG <10 (mg/ml)
A	54	11.1	25.3±10.2^a^	65.9±13.4^ab^	7 (13)	6 (11.1)
B	36	75	17.3±6.5^bcd^	53.1±6.1^de^	9 (25)	7 (19.4)
C	48	93.8	19.2±8.0^bcd^	53.9±6.7^de^	7 (14.6)	4 (8.3)
D	51	0	15.7 ± 9.3^cd^	52.6±7.1^de^	18 (35.3)	15 (29.4)
E	48	2.1	13.1±10.6^d^	48.8±8.5^e^	18 (37.5)	18 (37.5)
F	39	100	20.4±7.4^abc^	57.6±5.3^cd^	7 (17.9)	6 (15.4)
G	35	8.6	16.2 ± 8.9^cd^	54.1±7.1^de^	9 (25.7)	8 (22.9)
H	54	0	23.3±10.4^ab^	61.6±8.8^bc^	7 (13)	7 (13)
I	56	83.9	14.1±6.9^d^	51.7±5.5^e^	21 (37.5)	14 (25)
J	26	19.2	13.8±10.7^d^	52.8±8.0^de^	9 (34.6)	8 (30.8)
K	45	0	26.6±8.2^a^	67.4±10.4^a^	4 (8.9)	4 (8.9)
Total	492	35.2	19.0±10.0	56.7±10.3	116 (23.6)	97 (19.7)

*Values are mean±sd. Within columns, farms with different superscripts differed significantly on ANOVA testing a>b> c>d > e, P<0.05.

ANOVA, analysis of variance.

**FIG 2:**
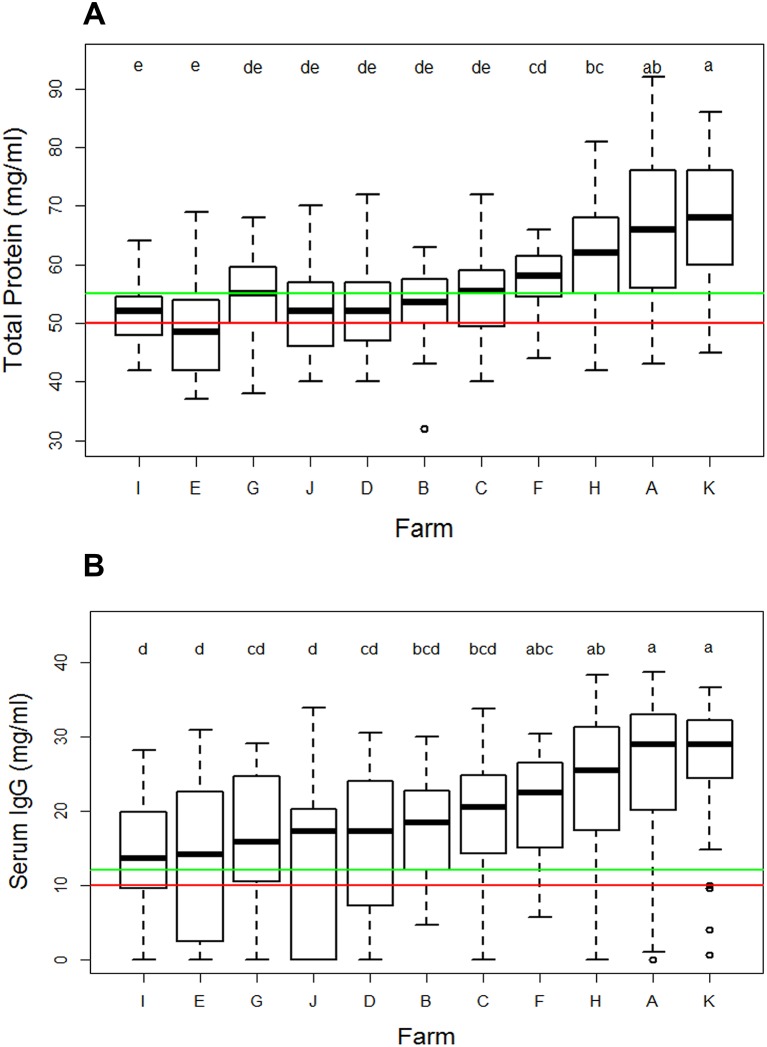
Box and whisker plots showing (A) serum IgG measured by radial immunodiffusion and (B) serum total protein measured by refractometer. Samples were taken at calf recruitment, from 1 to 8 days after birth. Results are split by farm of origin and farms are ordered by median value. Horizontal lines indicate the commonly used reference values: in (A) red is at 10 mg/ml and green at 12 mg/ml and in (B) red is at 50 mg/ml and green at 55 mg/ml. Letters show the results from analysis of variance: groups with the same letter did not differ significantly (P>0.05).

The mean serum IgG in the first week of life was more than 10 mg/ml on all farms, indicating adequate passive transfer (APT), with an overall mean of 19.0±10 mg/ml IgG (Table 3). The variation within farms is illustrated in Fig 2. The three farms with autumn block calving (A, H, K) had high mean IgG and a low number of calves with FPT, although they only supplemented calves rarely. Three farms had more than 25 per cent calves with less than10 mg/ml IgG. The overall mean value for TP was 56.7±10.3 mg/ml. One farm had an average TP of 48.8±8.5 mg/ml, below the accepted threshold for APT of more than 50 mg/ml. Overall, 20.7% of calves were recorded as having FPT when classified using IgG less than 10 mg/ml and 24.4 per cent using TP less than 50 mg/ml. As described in the ‘Introduction’ section, a range of cut-off points has been recommended previously. There was a large difference between the two methods in classifying calves with marginal results, with 4.1 per cent of calves having an IgG of 10–12 mg/ml but 21 per cent having a TP of 50–55 mg/ml.

### Calf mortality

For the cohort as a whole, 3.1 per cent of calves died in the first month and 1.4 per cent in the second, giving an overall mortality rate of 22/492 (4.5 per cent) (Table 4). The main causes of death were diarrhoea (n=8, all in the first month) or BRD (n=7, three in the first and four in the second month). Other causes were dystocia-related (n=2), omphalophlebitis (n=1), neonatal pancytopenia associated with BVD vaccination of the dam (n=1), rumen bloat (n=2) and accident (n=1). All farms had at least one death among their preweaned calves, ranging from 1 to 6. Of the total diagnosed cases for diarrhoea and BRD, 2.6 per cent (8/308) and 1.8 per cent (7/396) died, respectively.

**TABLE 4:** Duration and frequency of diarrhoea and bovine respiratory disease in preweaned calves based on once-weekly assessment using a modified Wisconsin-Madison health scoring system*

	A	B	C	D	E	F	G	H	I	J	K	Total n/%
n calves	54	36	48	51	48	39	35	54	56	26	45	492
n deaths (%)	2 (3.7)	3 (8.3)	3 (6.3)	2 (3.9)	2 (4.2)	1 (2.6)	1 (2.9)	1 (1.9)	4 (7.1)	2 (7.7)	1 (2.2)	22 (4.5)
Diarrhoea												
0 w	41	19	19	22	30	10	20	30	27	11	26	255
1 w	12	15	22	23	16	18	13	15	20	10	16	180
2–3 w	1	2	7	6	1	9	2	8	9	5	3	53
4–6 w	0	0	0	0	1	2	0	1	0	0	0	4
% healthy	75.9	52.8	39.6	43.1	62.5	25.6	57.1	55.6	48.2	42.3	57.8	51.8%
% sick ≥2 w	1.8	5.5	14.6	11.8	4.2	28.2	5.7	16.7	16.1	19.2	6.7	11.6%
% sick total	24.1	47.2	60.4	56.9	37.5	74.4	42.9	44.4	51.8	57.7	42.2	48.2%
BRD												
0 w	43	22	32	18	18	28	21	35	21	18	10	266
1 w	10	10	12	13	18	4	11	12	16	3	19	128
2–3 w	1	3	4	15	12	6	3	7	15	5	12	83
4–6 w	0	1	0	5	0	1	0	0	4	0	4	15
% healthy	79.6	61.1	66.7	35.3	37.5	71.8	60.0	64.8	37.5	69.2	22.2	54.1%
% sick ≥2 w	5.6	11.1	8.3	39.2	25.0	17.9	8.5	13.0	33.9	19.2	35.6	19.9%
% sick total	20.4	38.9	33.3	64.7	62.5	28.2	40.0	35.2	62.5	30.8	77.8	45.9%

*Each recruited calf is counted once. Healthy animals are recorded as having 0 weeks with the disease.

### Treatment of preweaning disease

Calves showing signs of clinical disease at each weekly examination were reported to the farmer and their veterinary surgeon and treated according to an established protocol as described above. It was not therefore possible to assess any treatment effects since all sick calves were treated. Farm I used a routine treatment with a long-acting macrolide antibiotic, for example, tilmicosin (Micotil, Elanco, Basingstoke, Hampshire, UK) or tulathromycin (Draxxin, Zoetis, London, UK) when calves were moved at 10 days old, in an attempt to reduce BRD. Veterinary surgeons were called out to treat animals at six farms on 10 occasions, three for group problems (one diarrhoea, Farm H) and two BRD outbreaks (Farms I, K). Five individual animals were treated by their veterinary surgeon for diarrhoea (n=2), BRD, neonatal pancytopenia and an umbilical injury.

### Incidence of diarrhoea

**FIG 3:**
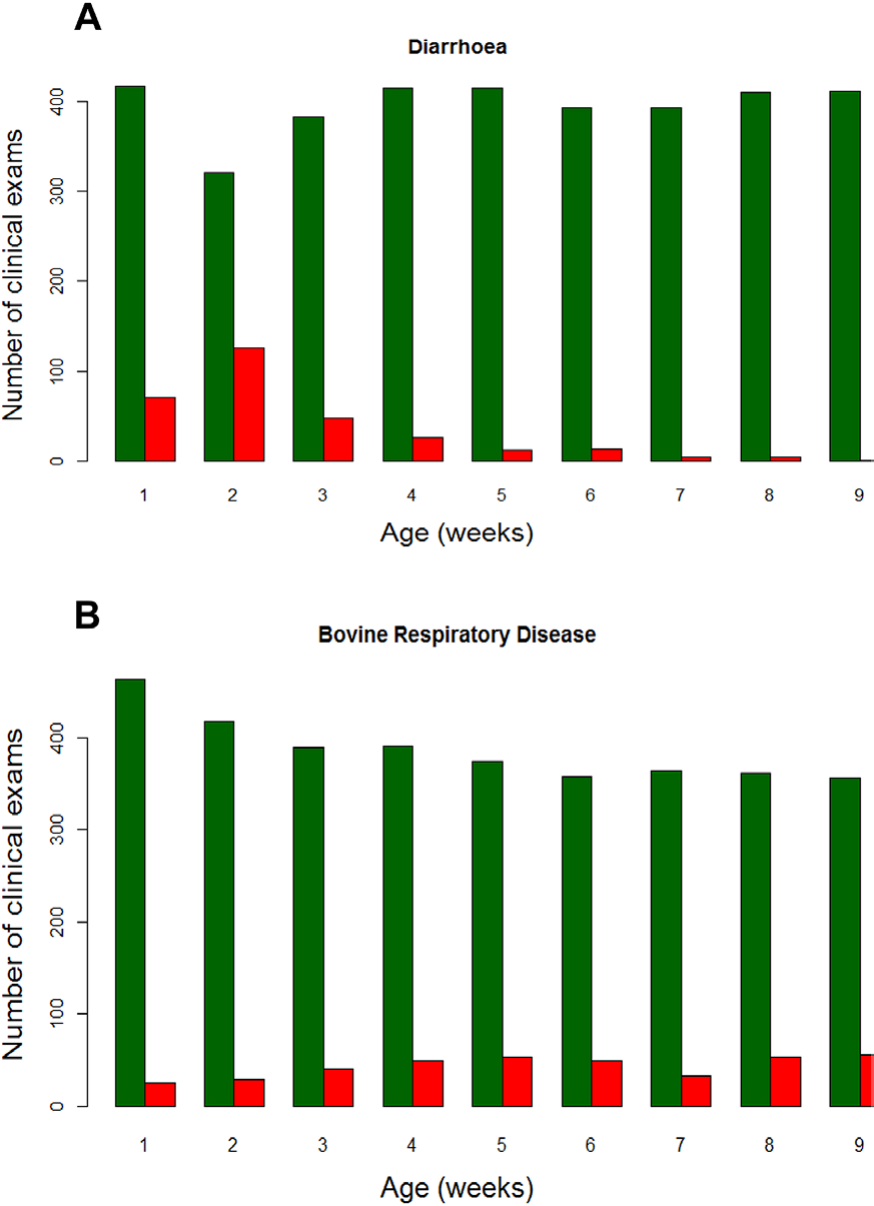
Number of clinical examinations performed on all 492 calves between recruitment and nine weeks of age. The green bars represent the number of examinations where calves were unaffected by disease and the red bars show the number of calves showing signs of (A) diarrhoea and (B) bovine respiratory disease based on assessment using a modified Wisconsin Madison calf health scoring system.

Throughout the whole preweaning period, 48.2 per cent of calves were diagnosed with diarrhoea with an overall incidence rate of 7.8 cases/100 calf weeks at risk (95 per cent CI: 7.1 to 8.8 per cent). The highest proportion of examinations where a calf showed signs of diarrhoea was at the second clinical examination, when 126 calves (28.2%) reached the threshold for diagnosis (Fig 3A). Very few diarrhoea cases were observed in calves over 4 weeks old, with less than 3% of older individuals affected in any week. There was a wide range of disease rates from 24.1% to 74.4% on different farms (Table 4). While the highest incidence (74.4 per cent) was on one of the larger farms (F), there was no overall relationship with herd size (ANOVA, P=0.26). Most calves showed clinical signs for one week, with only 24.0 per cent of calves affected for more than one week. On Farm F, however, 11/29 (37.9 per cent) of the calves with diarrhoea showed clinical signs for greater than and equal to2 weeks. Faecal scoring was used as the only method of diagnosing diarrhoea, but calves with diarrhoea had a higher mean temperature than those with normal faeces (healthy, 38.8^o^C (95  per cent CI: 38.78 to 38.89) vs diarrhoea, 39.01^o^C (95 per cent CI: 38.95 to 39.07), P<0.001). Overall, calves had a temperature more than 39.0^o^C (defined as pyrexia by McGuirk^1^ on 177/307 (57.6  per cent) of occasions where diarrhoea was detected.

### Incidence of BRD

BRD showed a different pattern, with cases spread throughout the preweaning period (Fig 3B). The incidence rate was 10.1 cases/100 calf weeks at risk (95per cent CI: 9.2 to 11.2 per cent). The smallest number of cases was observed in the first week, when 5.1 per cent of clinical examinations were positive. Cases of disease were high throughout the second month with more than 10 per cent of individuals affected in weeks four to nine. BRD of any duration was diagnosed in 45.9 per cent of calves (95 per cent CI: 41.2 to 50.3 per cent). There was a large variation in the proportion of calves affected between farms, ranging from 20.4 to 77.8 per cent (Table 4) and this was not related to herd size (ANOVA, P=0.14). Four farms had more than 60 per cent of calves showing BRD preweaning. Of those calves affected, 43.4 per cent showed clinical signs for more than one week. Farm J, with the least number of calves affected, also had the shortest duration of disease with 10/11 (90.9 per cent) individuals only showing signs of BRD for a single clinical examination. In contrast, only 28.2 per cent of calves on Farm F were affected but these had the longest duration of disease with 7/11 (63.6 per cent) showing clinical signs for two or more weeks. In calves diagnosed with BRD, 83.3 per cent had a rectal temperature greater than and equal to 39°C. The mean rectal temperatures were significantly different (P<0.001): healthy, 38.8^o^C (95 per cent CI: 38.73 to 38.83) vs BRD, 39.4^o^C (95 per cent CI: 38.34 to 39.44).

## Discussion

This study has quantified passive transfer, health and mortality in preweaned dairy heifers on 11 commercial UK dairy farms. These were all typical commercial farms, although they were relatively large for the UK, ranging in size from 186 to 550 adult cows, compared with the UK average of 133.^29^  Calf management was similar to that seen in a stratified sample of 102 UK dairy herds.^8^ This was rarely a specialised task and generally involved several farm staff. There were few centralised methods of data recording for this age group and, as has been shown in other studies,^28^ there was little recording of disease in heifer calves by farmers. Calf health has been considered here at a farm level rather than an individual calf level; risk factors for disease in individual calves will form the basis of the next paper. In practice, calves are managed as groups with farmers and their veterinary surgeons making most herd health and management decisions based on group performance.

Disease cases are affected by a triad of factors and their interactions: the environment, the pathogen and the calf. When considering a cohort born on the same farm at a similar time of year, two of these three factors remain reasonably constant. Disease was assessed using an adapted Wisconsin-Madison Scoring system, which is now widely established as a useful systematic way to determine calf health.^1^ Clinical scoring could be undertaken easily and quickly by one person and standardised definitions of disease allow comparison with other studies. This method was not, however, a good tool to discriminate between upper respiratory tract infections and pneumonia. Additionally, for both BRD and diarrhoea, the weekly assessment interval used means that some cases recorded as continuing cases could potentially have been relapses. The influence of disease treatments could not be investigated here as all sick animals were treated promptly according to standardised protocols.

The incidence of mortality among heifers which were born alive but died in their first month was similar to that reported in a previous UK cohort, at 3.1 per cent compared with 3.4 per cent^2^; mortality in calves in the second month of life was lower at 1.4 per cent. Diarrhoea and BRD each accounted for about one-third of the deaths. All incidences of disease were reported to the farm and the veterinarian on a weekly basis, with comparative overall results between farms reported both midway through and at the end of the study for benchmarking purposes. The weekly reports might potentially have improved diagnosis and treatment and so reduced the fatality rate.

A previous UK study found that 26 per cent of 444 calves tested had FPT (range 5–51 per cent between seven farms tested,^32^ compared with 20.7 per cent in this cohort. In comparison, a much higher proportion of 48 per cent of 414 Welsh calves sampled had FPT based on zinc sulphate turbidity testing (ZST).^28^ Beam and others^33^ undertook a large-scale assessment of colostrum management in the USA and found that 19.2 per cent of calves experienced FPT even though 76 per cent were reported to receive supplementary colostrum. In the present study, there was a much lower intervention rate of 35.2 per cent with a mean IgG of 19 mg/ml. There was, however, high variability between farms for mean IgG and TP results. Farms with delayed cow/calf separation and autumn block calving had better passive transfer than farms that separated calves immediately and had AYR calving patterns (Table 3). This suggests a possible conflict between Johne’s disease management, where disease spread can be reduced by early separation and feeding heat-treated colostrum,^34^ against the good passive transfer that appears to occur when cow and calf are uninterrupted. More tailored advice may therefore be useful for farmers, depending on their Johne’s disease status.

Despite the good passive transfer, contagious disease was highly prevalent, with 48 per cent of calves diagnosed with diarrhoea and 46 per cent with BRD. The farms included in the study were not a random sample. It is therefore possible there was bias in farm selection, although no disease-related criteria were used for inclusion. Another recent UK cross-sectional study also found a high disease incidence, but only 39 per cent of farmers were recording calf disease, meaning that high rates may be going unnoticed.^28^ Diarrhoea incidence was higher than that reported in most other cohorts on commercial farms in other countries, which ranged from 10 to 35 per cent (reviewed by Johnson and others^4^). One study with a higher incidence of 64 per cent deliberately recruited farms with a known diarrhoea problem: diagnosis was based initially on clinical signs and samples were then taken for pathogen identification.^35^ In the present study, diarrhoea diagnosis was only based on clinical signs so it is possible that some of the cases were non-contagious diet-related scours. Diarrhoea can also be associated with rumen drinking in bucket-fed calves.^36^ Milk may enter the rumen due to failure of closure of the oesophageal groove causing acidosis and inflammation and potentially leading to rumen ulceration and secondary bacterial and fungal infection. However, samples from 91.2 per cent of diarrhoeic calves in another study tested positive for enteric pathogens.^37^ Another limitation of the current study was that all the calves recruited were born between August and February. This coincided with peak calving periods but did mean that spring and summer births were omitted. Season is assumed to be an important factor in calf disease, although there is conflicting evidence as to when higher disease and mortality rates may be expected.^11 38^ Some studies on research farms have included all calves born over several years.^13^ It is rare that this can be achieved on commercial farm, although future work should address this question of seasonality in the UK.

BRD was more common than in any recently reported cohort, which reported incidences ranging from 1 to 39 per cent.^4^  Comparing two separate studies in New York State, USA, the reported incidence of BRD was 21.6 per cent higher when it was recorded by veterinarians^10^ compared with farmers.^39^  The high incidence found here may reflect the relatively intensive monitoring regime used in the current study, for example, compared with less frequent visits^40^ or with farmer reporting.^14^ Disease was usually first diagnosed at the clinical examination rather than by farmers. This is to be expected as farmers often use reduced milk consumption as the main sign of ill health and, when calves are fed limited rations, this is a late sign of disease.^27^ The diagnostic criteria used for both diarrhoea and BRD were nevertheless similar to several previous studies, suggesting that the disease rates on these UK farms really were comparatively high.

Of calves with BRD, almost half had clinical signs for longer than one week including pyrexia. This is associated with changes in behaviour and poor welfare through the expression of sickness behaviours^41^ suggesting that the high rates of BRD recorded indicate potential problems for calf welfare. Once contagious disease has occurred, there is increased risk of future morbidity: previous diarrhoea was associated with repeated loose faeces^42 43^ and later BRD.^39 42 44^

Diarrhoea in the first month was the single most frequent cause of death in this cohort, despite good passive transfer. Recent literature has suggested that there is commonly no significant relationship between contracting diarrhoea and passive transfer measured in blood. For example, only 10.3 per cent of calves had a serum TP less than 52 mg/ml in a recent Canadian study on farms with an existing diarrhoea problem.^35^ Similar large cohort studies of 200–600 heifers also found no correlation.^10 14 17 35^ In another recent cohort of 2874 heifers recruited from 19 commercial dairy farms, failure of passive transfer was not associated with neonatal diarrhoea when other factors were accounted for in the analysis.^26^ A complexity of investigating this type of relationship is that the pathological effects of diarrhoea can cause either dehydration or protein loss via the gut, so TP may be abnormally high or low in diarrhoeic calves. In the present study, serum IgG was recorded in addition to TP in an attempt to mitigate this problem. Increasing use of serum total protein to monitor passive transfer^24 35^ supports the need for further study on an individual calf basis to explore this relationship.

There was regularly insufficient availability of water on some farms despite a legal requirement to provide water to calves aged over one week. This generally occurred when buckets were spilled or fouled, but was also observed when calves were late in transfer to a rearing unit. In a previous survey of 102 UK dairy farms, only 78 per cent of farms provided water to calves within seven days of birth and two farms did not provide water until after weaning.^8^ It was noticeable that diarrhoea typically occurred in very young calves which generally had little access to water. A lack of water, especially if associated with a restricted milk ration, would worsen dehydration and contribute to mortality. Most calf diarrhoea is contagious in origin.^35 37^ Therefore, to prevent diarrhoea, farmers should be advised to focus on improving hygiene of housing and feeding. Although cleanliness has excellent evidence to support its benefits,^4 19^ this strategy was recently reported to be a low priority among Welsh farmers.^28^

Analysis of results on disease incidence with respect to herd size have varied, and no associations were observed in this study. In previous studies, the risks of more calf mortality^3 45^ *Cryptosporidium  parvum* shedding^46^ and the number of cases of BRD diagnosed^44^ were all higher in larger herds. However, in an extensive study of around 47 000 heifers in the USA, farm size showed no association with mortality and lower producing units (which tended to be smaller) had higher mortality.^47^ It is possible that herd size can interact with other factors explaining the associations found in some previous reports. This will be examined in the next paper on this cohort to determine the main risk factors for disease on an individual calf basis.

## Conclusion

In summary, this cohort of UK heifers performed fairly well in some areas: passive transfer was better than or similar to other cohorts of heifers studied, with only 21 per cent of animals having IgG less than 10 mg/ml. The mortality rate of 4.5 per cent was also relatively low. This cohort nevertheless performed worse than those studied internationally with respect to the incidence of contagious endemic disease with very high rates of both BRD and diarrhoea. Although this did not appear to cause high mortality, disease does indicate poor calf welfare. There were also clear needs for improved provision of clean water for any sick animal and all calves over seven days old, to meet the legal requirement. In order to minimise disease in their stock, farmers need additional training in prompt recognition and recording the signs of disease and they should also be encouraged to monitor their mortality rates. They can then work with their veterinary surgeons to use this knowledge to optimise their general management strategies and to develop protocols to identify and respond to disease appropriately.

## References

[1] McGuirk SM Disease management of dairy calves and heifers. *Veterinary Clinics of North America* . Food Animal Practice 2008;24:139–53. 18299036 10.1016/j.cvfa.2007.10.003 PMC7135781

[2] Brickell JS, McGowan MM, Pfeiffer DU, et al Mortality in Holstein-Friesian calves and replacement heifers, in relation to body weight and IGF-I concentration, on 19 farms in England. Animal 2009;3:1175–82. doi:10.1017/S175173110900456X 22444847 10.1017/S175173110900456X

[3] Gulliksen SM, Lie KI, Løken T, et al Calf mortality in Norwegian dairy herds. J Dairy Sci 2009;92:2782–95. doi:10.3168/jds.2008-1807 19448012 10.3168/jds.2008-1807

[4] Johnson KF, Burn CC, Wathes DC Rates and risk factors for contagious disease and mortality in young dairy heifers. CAB Reviews 2011;6:059 doi:10.1079/PAVSNNR20116059

[5] Sivula NJ, Ames TR, Marsh WE Management practices and risk factors for morbidity and mortality in Minnesota dairy heifer calves. Prev Vet Med 1996;27:173–82. doi:10.1016/0167-5877(95)01001-7

[6] Torsein M, Lindberg A, Sandgren CH, et al Risk factors for calf mortality in large Swedish dairy herds. Prev Vet Med 2011;99:136–47. doi:10.1016/j.prevetmed.2010.12.001 21257214 10.1016/j.prevetmed.2010.12.001 PMC7132482

[7] Johnson RW The concept of sickness behavior: a brief chronological account of four key discoveries. Vet Immunol Immunopathol 2002;87:443–50. doi:10.1016/S0165-2427(02)00069-7 12072271 10.1016/s0165-2427(02)00069-7

[8] Boulton AC, Rushton J, Wathes DC A study of dairy heifer rearing practices from birth to weaning and their associated costs on uk dairy farms. Open J Anim Sci 2015;05:185–97. doi:10.4236/ojas.2015.52021

[9] Waltner-Toews D, Martin SW, Meek AH The effect of early calfhood health status on survivorship and age at first calving. Can J Vet Res 1986;50:314–7. 3742366 PMC1255219

[10] Virtala AM, Mechor GD, Gröhn YT, et al The effect of calfhood diseases on growth of female dairy calves during the first 3 months of life in New York State. J Dairy Sci 1996;79:1040–9. doi:10.3168/jds.S0022-0302(96)76457-3 8827469 10.3168/jds.S0022-0302(96)76457-3 PMC7130866

[11] Svensson C, Liberg P The effect of group size on health and growth rate of Swedish dairy calves housed in pens with automatic milk-feeders. Prev Vet Med 2006;73:43–53. doi:10.1016/j.prevetmed.2005.08.021 16191449 10.1016/j.prevetmed.2005.08.021

[12] Correa MT, Curtis CR, Erb HN, et al Effect of calfhood morbidity on age at first calving in New York Holstein herds. Prev Vet Med 1988;6:253–62. doi:10.1016/0167-5877(88)90037-2

[13] Bach A Associations between several aspects of heifer development and dairy cow survivability to second lactation. J Dairy Sci 2011;94:1052–7. doi:10.3168/jds.2010-3633 21257075 10.3168/jds.2010-3633

[14] Sivula NJ, Ames TR, Marsh WE, et al Descriptive epidemiology of morbidity and mortality in Minnesota dairy heifer calves. Prev Vet Med 1996;27:155–71. doi:10.1016/0167-5877(95)01000-9

[15] Svensson C, Lundborg K, Emanuelson U, et al Morbidity in Swedish dairy calves from birth to 90 days of age and individual calf-level risk factors for infectious diseases. Prev Vet Med 2003;58:179–97. doi:10.1016/S0167-5877(03)00046-1 12706057 10.1016/s0167-5877(03)00046-1

[16] Scott PR, Hall GA, Johnes PW, et al Bovine medicine: diseases and husbandry of cattle : Bovine medicine: diseases and husbandry of cattle. 2nd edn Hoboken, NJ: Wiley-Blackwell, 2008:185–215.

[17] Donovan GA, Dohoo IR, Montgomery DM, et al Associations between passive immunity and morbidity and mortality in dairy heifers in Florida, USA. Prev Vet Med 1998;34:31–46. doi:10.1016/S0167-5877(97)00060-3 9541949 10.1016/S0167-5877(97)00060-3 PMC7134088

[18] Gulliksen SM, Jor E, Lie KI, et al Enteropathogens and risk factors for diarrhea in Norwegian dairy calves. J Dairy Sci 2009;92:5057–66. doi:10.3168/jds.2009-2080 19762824 10.3168/jds.2009-2080 PMC7094401

[19] Vasseur E, Borderas F, Cue RI, et al A survey of dairy calf management practices in Canada that affect animal welfare. J Dairy Sci 2010;93:1307–16. doi:10.3168/jds.2009-2429 20172250 10.3168/jds.2009-2429

[20] Patel S, Gibbons J, Wathes DC Ensuring optimal colostrum transfer to new-born dairy calves. Cattle Practice 2014;22:95–104.

[21] Korhonen H, Marnila P, Gill HS Milk immunoglobulins and complement factors. Br J Nutr 2000;84(Suppl 1):S75–80. doi:10.1017/S0007114500002282 11242450 10.1017/s0007114500002282

[22] Weaver DM, Tyler JW, VanMetre DC, et al Passive transfer of colostral immunoglobulins in calves. J Vet Intern Med 2000;14:569–77. doi:10.1111/j.1939-1676.2000.tb02278.x 11110376 10.1892/0891-6640(2000)014<0569:ptocii>2.3.co;2

[23] Hammer CJ, Quigley JD, Ribeiro L, et al Characterization of a colostrum replacer and a colostrum supplement containing IgG concentrate and growth factors. J Dairy Sci 2004;87:106–11. doi:10.3168/jds.S0022-0302(04)73147-1 14765816 10.3168/jds.S0022-0302(04)73147-1

[24] Godden S Colostrum management for dairy calves. Vet Clin North Am Food Anim Pract 2008;24:19–39. doi:10.1016/j.cvfa.2007.10.005 18299030 10.1016/j.cvfa.2007.10.005 PMC7127126

[25] Trotz-Williams LA, Leslie KE, Peregrine AS Passive immunity in Ontario dairy calves and investigation of its association with calf management practices. J Dairy Sci 2008;91:3840–9. doi:10.3168/jds.2007-0898 18832206 10.3168/jds.2007-0898

[26] Windeyer MC, Leslie KE, Godden SM, et al Factors associated with morbidity, mortality, and growth of dairy heifer calves up to 3 months of age. Prev Vet Med 2014;113:231–40. doi:10.1016/j.prevetmed.2013.10.019 24269039 10.1016/j.prevetmed.2013.10.019

[27] Svensson C, Jensen MB Short communication: Identification of diseased calves by use of data from automatic milk feeders. J Dairy Sci 2007;90:994–7. doi:10.3168/jds.S0022-0302(07)71584-9 17235177 10.3168/jds.S0022-0302(07)71584-9

[28] Atkinson O To survey current practices and performance and to determine the success factors for rearing replacement dairy heifers in Wales. The Welsh Dairy Youngstock Project, full report. 2015 http://dairyveterinaryconsultancy.co.uk/download/the-welsh-dairy-youngstock-project-full-report/ (accessed 22 Jul 2017).

[29] Bate A UK dairy statistics house of commons briefing paper; 2016, Report No: 2721 http://researchbriefings.files.parliament.uk/documents/SN02721/SN02721.pdf

[30] Brickell JS, McGowan MM, Wathes DC Effect of management factors and blood metabolites during the rearing period on growth in dairy heifers on UK farms. Domest Anim Endocrinol 2009;36:67–81. doi:10.1016/j.domaniend.2008.10.005 19059748 10.1016/j.domaniend.2008.10.005

[31] van der Burgt G, Hepple S Legal position on ‘once a day’ feeding of artificial milk to calves. Vet Rec 2013;172:135 doi:10.1136/vr.f623 23378312 10.1136/vr.f623

[32] MacFarlane JA, Grove-White DH, Royal MD, et al Identification and quantification of factors affecting neonatal immunological transfer in dairy calves in the UK. Vet Rec 2015;176:625 doi:10.1136/vr.102852 25861824 10.1136/vr.102852

[33] Beam AL, Lombard JE, Kopral CA, et al Prevalence of failure of passive transfer of immunity in newborn heifer calves and associated management practices on US dairy operations. J Dairy Sci 2009;92:3973–80. doi:10.3168/jds.2009-2225 19620681 10.3168/jds.2009-2225

[34] Stabel JR On-farm batch pasteurization destroys Mycobacterium paratuberculosis in waste milk. J Dairy Sci 2001;84:524–7. doi:10.3168/jds.S0022-0302(01)74503-1 11233038 10.3168/jds.S0022-0302(01)74503-1

[35] Trotz-Williams LA, Wayne Martin S, Leslie KE, et al Calf-level risk factors for neonatal diarrhea and shedding of Cryptosporidium parvum in Ontario dairy calves. Prev Vet Med 2007;82:12–28. doi:10.1016/j.prevetmed.2007.05.003 17602767 10.1016/j.prevetmed.2007.05.003 PMC7114353

[36] Van Weeren-Keverling Buisman A, Mouwen JM, Wensing T, et al Intraruminal administration of milk in the calf as a model for ruminal drinking: morphological and enzymatical changes in the jejunal mucosa. Vet Res Commun 1990;14:129–40. doi:10.1007/BF00346553 2161140 10.1007/BF00346553 PMC7089113

[37] Uhde FL, Kaufmann T, Sager H, et al Prevalence of four enteropathogens in the faeces of young diarrhoeic dairy calves in Switzerland. Vet Rec 2008;163:362–6. doi:10.1136/vr.163.12.362 18806281 10.1136/vr.163.12.362

[38] Hamnes IS, Gjerde B, Robertson L Prevalence of giardia and cryptosporidium in dairy calves in three areas of norway. Vet Parasitol 2006;140:204–16. doi:10.1016/j.vetpar.2006.03.024 16647210 10.1016/j.vetpar.2006.03.024

[39] Curtis CR, Erb HN, White ME Descriptive epidemiology of calfhood morbidity and mortality in New York holstein herds. Prev Vet Med 1988;5:293–307. doi:10.1016/0167-5877(88)90015-3

[40] Windeyer MC, Leslie KE, Godden SM, et al Association of bovine respiratory disease or vaccination with serologic response in dairy heifer calves up to three months of age. Am J Vet Res 2015;76:239–45. doi:10.2460/ajvr.76.3.239 25710760 10.2460/ajvr.76.3.239

[41] Broom DM Behaviour and welfare in relation to pathology. Appl Anim Behav Sci 2006;97:73–83. doi:10.1016/j.applanim.2005.11.019

[42] Perez E, Noordhuizen JPTM, van Wuijkhuise LA, et al Management factors related to calf morbidity and mortality rates. Livestock Production Science 1990;25:79–93. doi:10.1016/0301-6226(90)90043-6

[43] Bartels CJ, Holzhauer M, Jorritsma R, et al Prevalence, prediction and risk factors of enteropathogens in normal and non-normal faeces of young Dutch dairy calves. Prev Vet Med 2010;93:162–9. doi:10.1016/j.prevetmed.2009.09.020 19819574 10.1016/j.prevetmed.2009.09.020 PMC7125667

[44] Van Donkersgoed J, Ribble CS, Boyer LG, et al Epidemiological study of enzootic pneumonia in dairy calves in Saskatchewan. Can J Vet Res 1993;57:247–54. 8269363 PMC1263636

[45] Lance SE, Miller GY, Hancock DD, et al Effects of environment and management on mortality in preweaned dairy calves. J Am Vet Med Assoc 1992;201:1197–202. 1429158

[46] Trotz-Williams LA, Martin SW, Leslie KE, et al Association between management practices and within-herd prevalence of Cryptosporidium parvum shedding on dairy farms in southern Ontario. Prev Vet Med 2008;83:11–23. doi:10.1016/j.prevetmed.2007.03.001 17481752 10.1016/j.prevetmed.2007.03.001 PMC7114088

[47] Losinger WC, Heinrichs AJ Factors associated with high scours mortality among preweaned dairy heifers. Archivos de Zootecnia 1997;46:311–22. 10.1017/s0022029996001999 9120071

